# RON5 Is Critical for Organization and Function of the *Toxoplasma* Moving Junction Complex

**DOI:** 10.1371/journal.ppat.1004025

**Published:** 2014-03-20

**Authors:** Josh R. Beck, Allan L. Chen, Elliot W. Kim, Peter J. Bradley

**Affiliations:** Department of Microbiology, Immunology and Molecular Genetics, University of California, Los Angeles, Los Angeles, California, United States of America; MRC National Institute for Medical Research, United Kingdom

## Abstract

Apicomplexans facilitate host cell invasion through formation of a tight-junction interface between parasite and host plasma membranes called the moving junction (MJ). A complex of the rhoptry neck proteins RONs 2/4/5/8 localize to the MJ during invasion where they are believed to provide a stable anchoring point for host penetration. During the initiation of invasion, the preformed MJ RON complex is injected into the host cell where RON2 spans the host plasma membrane while RONs 4/5/8 localize to its cytosolic face. While much attention has been directed toward an AMA1-RON2 interaction supposed to occur outside the cell, little is known about the functions of the MJ RONs positioned inside the host cell. Here we provide a detailed analysis of RON5 to resolve outstanding questions about MJ complex organization, assembly and function during invasion. Using a conditional knockdown approach, we show loss of RON5 results in complete degradation of RON2 and mistargeting of RON4 within the parasite secretory pathway, demonstrating that RON5 plays a key role in organization of the MJ RON complex. While RON8 is unaffected by knockdown of RON5, these parasites are unable to invade new host cells, providing the first genetic demonstration that RON5 plays a critical role in host cell penetration. Although invasion is not required for injection of rhoptry effectors into the host cytosol, parasites lacking RON5 also fail to form evacuoles suggesting an intact MJ complex is a prerequisite for secretion of rhoptry bulb contents. Additionally, while the MJ has been suggested to function in egress, disruption of the MJ complex by RON5 depletion does not impact this process. Finally, functional complementation of our conditional RON5 mutant reveals that while proteolytic separation of RON5 N- and C-terminal fragments is dispensable, a portion of the C-terminal domain is critical for RON2 stability and function in invasion.

## Author Summary

*Toxoplasma* and related apicomplexan parasites are obligate intracellular pathogens that actively invade their host cells, creating a specialized vacuole within which the parasite is able to replicate. Invasion involves the establishment of a tight-junction interface between host and parasite membranes called the moving junction (MJ) through which the parasite actively penetrates the host. At the onset of invasion, a protein complex composed of RONs 2/4/5/8 is injected from specialized parasite secretory organelles called rhoptries into the host membrane. Following secretion, this RON complex localizes to the MJ throughout the invasion event and is thought to be the basis for this tight-junction. In this study, we utilize a conditional knockdown of RON5 to show that this MJ component, present at the cytosolic face of the host membrane during penetration, is crucial for invasion and for MJ complex organization. In particular, loss of RON5 results in degradation of RON2 and mistargeting of RON4 in the parasite, effectively ablating the MJ complex. We exploit this knockdown strain to evaluate RON5 processing and identify regions of the protein that are necessary for organizing the complex. Our findings demonstrate the key role of RON5 in facilitating apicomplexan host invasion and disease.

## Introduction

The Apicomplexa are a large phylum of eukaryotic pathogens comprised of ∼6,000 described species which cause extensive disease in humans and other animals [1], [2]. Species of particular interest include *Toxoplasma gondii*, which chronically infects approximately one-third of all humans and causes neurological disorders in immunocompromised individuals as well as the human malarial agent, *Plasmodium falciparum*, which is the cause of nearly a million deaths annually [3], [4]. The disease caused by these obligate intracellular parasites is dependent upon their ability to penetrate, form a specialized vacuole, and replicate within their host cells [5]. Thus, a better understanding of the parasite molecules and processes that facilitate host cell invasion is needed to aid in development of better therapeutics and control strategies.

Invasion in apicomplexans is a highly coordinated process of attachment and penetration that depends on sequential protein secretion events from two different organelles, the micronemes and rhoptries [6]. Initially, secretion from the micronemes releases molecular adhesins onto the parasite's plasma membrane, facilitating attachment to the host cell surface [7]. Translocation of these adhesins in an apical to posterior direction via an actin-myosin motor within the parasite pellicle generates a unique gliding motility which is thought to provide the force for host cell penetration. Intriguingly, the recent disruption of MIC2 and myosin A, key components of the gliding motility machinery previously considered essential to invasion, suggests the existence of alternative forces that can drive parasite penetration [8], [9].

After initial attachment, the parasite apex is oriented toward the host cell, followed by discharge of the rhoptry contents [10]. Rhoptry secretion corresponds with the formation of a ring-shaped tight-junction interface between parasite and host plasma membranes called the moving junction (MJ) through which the parasite passes to enter the host cell. A complex of the rhoptry neck proteins RONs 2/4/5/8 localizes to the MJ during invasion where it is thought to provide a stable anchoring point for host cell penetration, possibly through interaction with the host cell cytoskeleton as host cytoskeletal components localize to the MJ and are important for invasion [11]–[15]. The MJ is also the site of a molecular sieve that restricts access of host plasma membrane proteins to the nascent parasitophorous vacuole, rendering the vacuole non-fusogenic and protecting the parasite from lysosomal destruction, a function that may be performed by the MJ RON complex [16].

The micronemal adhesin AMA1 tightly binds RON2 in both *Toxoplasma* and *P. falciparum* extracts and peptides or antibodies which block this interaction interfere with invasion [11], [17]–[22]. These findings led to a model in which binding of RON2 (a transmembrane protein injected from the rhoptries into the host plasma membrane) to the ectodomain of AMA1 (a transmembrane protein secreted from the micronemes into the parasite plasma membrane) mediates tight-junction formation to bridge the invading parasite and host cell surfaces. However, the actual importance of the RON2-AMA1 interaction for tight-junction formation and invasion is now in question following recent reports showing that disruption of AMA1 has no detectible function in the MJ-mediated penetration step of invasion but instead plays a key role in adhesion [23], [24].

In contrast to RON2 and AMA1, RONs 4/5/8 are soluble proteins that are positioned in the host cytoplasm during invasion [13], [14]. A knockout of the coccidia-restricted MJ component RON8 shows that while not essential, this protein is important for efficient invasion [25]. Taken together with the conservation of the other MJ RONs across the Apicomplexa, these data suggest a core complex of RON2/4/5 comprises the critical invasion machinery. However, current genetic evidence for the role of this core complex in invasion is limited to a conditional RON4 mutant in *P. berghei* that inhibits invasion by sporozoites, highlighting the need for direct functional analysis of RON5 and RON2 by reverse genetics approaches [23].

Here we provide a comprehensive analysis of *Toxoplasma* RON5 to evaluate its role in assembly of the MJ complex and function in invasion. Using a conditional knockdown approach, we show that depletion of RON5 results in the complete loss of RON2 and mistargeting of RON4, indicating RON5 is critical for organization of the MJ complex. In contrast, targeting of RON8 is unaffected by disruption of the RON2/4/5 complex, in keeping with it being a coccidial-specific addition to the core complex. Parasites lacking RON5 egress efficiently but cannot invade new host cells or inject rhoptry effectors into the host cytosol, demonstrating the key importance of the MJ RON core complex in host cell penetration. Complementation of RON5 knockdown parasites with a series of mutants reveals that while proteolytic separation of RON5 N- and C-terminal fragments is dispensable, the C-terminal domain is critical for RON2 stability and MJ function. Together, this work demonstrates that RON5 is crucial for the organization of the MJ complex and provides the first genetic demonstration that the MJ RON core complex is critical for host cell invasion by the *Toxoplasma* parasite.

## Results

### An N-terminal domain of RON5 does not participate in the mature MJ complex

During maturation in transit to the rhoptries, RON5 is processed at least twice to separate the protein into three fragments. Antibodies raised against the RON5-N or -C fragments demonstrated that both were incorporated into the mature MJ complex and secreted into the MJ during invasion [13], [14]. However, the fate of the fragment removed by a more N-terminal processing event (predicted to be residues 34–314 following removal of the signal peptide) remains to be characterized. Several rhoptry proteins contain N-terminal pro-domains that are critical for organelle targeting and thus this region may constitute a pro-domain for trafficking of this component of the MJ complex [26]–[28]. Alternatively, this region may be incorporated into the mature complex and function in the MJ during invasion. A stretch of hydrophobic residues that could form a transmembrane domain is present in this region, and thus two models have been proposed for topology during invasion with RON5 either spanning the host plasma membrane similar to RON2 or soluble within the host cytosol [13], [14], [29].

**Figure 1:**
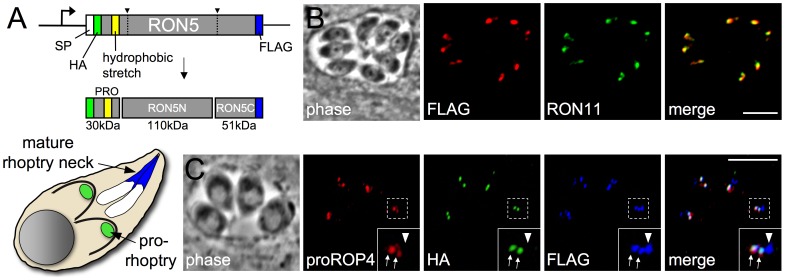
The N-terminus of RON5 is a pro domain that is not incorporated into the mature MJ complex. (A) Diagram showing RON5 double epitope tagging strategy. An HA tag was inserted inframe immediately downstream of the signal peptide and a FLAG tag was fused to the 3′ end of the coding sequence. This HA-N-RON5-C-FLAG second copy was targeted to the UPRT locus under the control of the endogenous RON5 promoter. A predicted hydrophobic stretch within the N-terminal region of RON5 is shown in yellow. (B) IFA showing RON5C-FLAG signal colocalizes with RON11 in the rhoptry neck. Red: mouse anti-FLAG antibody detected by Alexa594-anti-mouse IgG. Green: rat anti-RON11 antibody detected by Alexa488-anti-rat IgG. (C) IFA showing localization of the double epitope tagged RON5 protein during rhoptry maturation. The N-terminal portion of RON5 tagged with HA (green) co-localizes with proROP4 (red), indicating it is present in the pro-rhoptry compartment (arrows). RON5C-FLAG (blue) is present in the pro-rhoptry as well but also labels the mature rhoptries (arrowhead), visible as a distinct compartment anterior to the pro-rhoptries in each cell that does not contain proROP4. Importantly, the HA signal never co-localizes with the FLAG signal in the absence of proROP4, indicating it is not present in the mature rhoptries. Red: rabbit anti-proROP4 antibody detected by Alexa594-anti-rabbit IgG. Green: rat anti-HA antibody detected by Alexa488-anti-rat IgG. Blue: mouse anti-FLAG antibody detected by Alexa350-anti-mouse IgG. All scale bars = 5 µm.

To resolve this point, we generated a double epitope tagged version of RON5 with a FLAG tag at the C-terminus and an internal HA tag just downstream of the predicted signal peptide cleavage site (Figure 1A). This version of RON5 was placed under control of the RON5 promoter and the resulting expression cassette was targeted to the UPRT locus to enable stable expression of this double-tagged second copy of RON5. As expected, the C-terminal FLAG tag was readily detected in the rhoptry necks, as assessed by colocalization with the non-MJ rhoptry neck marker RON11 (Figure 1B). We then monitored rhoptry maturation in parasites expressing this cassette using an antibody against the pro-domain of ROP4 that specifically labels pro-rhoptry compartments [30]. While RON5C-FLAG was present in both pro and mature rhoptries, HA signal was only detected in proROP4-positive compartments, demonstrating that the N-terminal portion of RON5 is a pro-domain (hereafter referred to as proRON5) that is not present in mature rhoptries and thus is not incorporated into the mature MJ complex (Figure 1C).

### Establishment of a RON5 conditional knockdown strain

**Figure 2:**
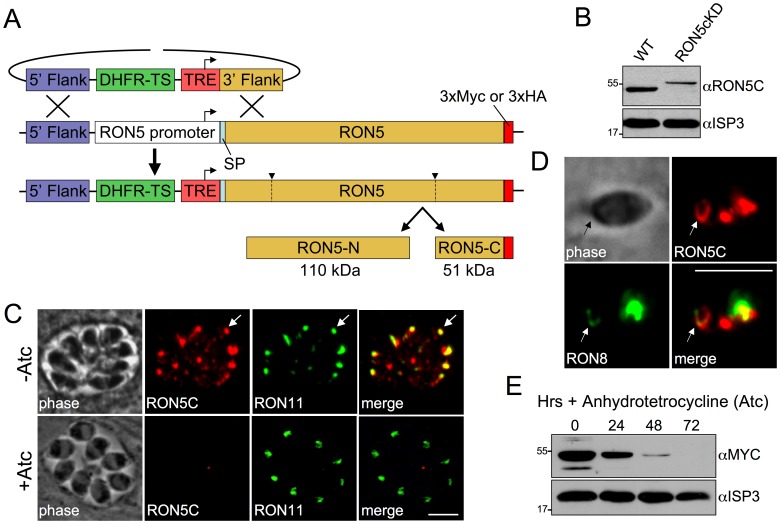
Establishment of a RON5 conditional knockdown mutant. (A) Strategy for generating RON5cKD parasites through direct replacement of the RON5 endogenous promoter with a tetracycline-repressible element (TRE) by homologous recombination. The TRE consists of seven tandem tetracycline operator sequences fused to a truncated SAG4 promoter. Prior to promoter exchange, a C-terminal endogenous 3xMYC or 3xHA tag was introduced at the C-terminus for improved detection. (B) Western blot comparing RON5C in wild-type and RON5_MYC_cKD strains without Atc. Exchange of the endogenous RON5 promoter with the TRE results in lower levels of basal RON5 expression. As expected, an ∼5 kD upshift in the RON5_MYC_cKD strain corresponding to the endogenous 3xMYC tag is also observed. The IMC protein ISP3 serves as a loading control. (C) IFA of intracellular RON5_HA_cKD parasites after 48 hours −/+ Atc. Some mistargeting of RON5C is seen in untreated parasites due to the replacement of the endogenous, cell cycle regulated promoter with the constitutive SAG4 promoter in the TRE. A major focus of RON5 signal still colocalizes with RON11 in the rhoptry neck (arrows). No gross impact on the rhoptries is observed following depletion of RON5 as assessed by the non-MJ rhoptry neck protein RON11. Red: rabbit anti-HA antibody detected by Alexa594-anti-rabbit IgG. Green: rat anti-RON11 antibody detected by Alexa488-anti-rat IgG. (D) IFA of an invading RON5_HA_cKD parasite. RON5C-3xHA signal is seen in the MJ (arrows) where it colocalizes with RON8. Red: rabbit anti-HA antibody detected by Alexa594-anti-rabbit IgG. Green: mouse anti-RON8 antibody detected by Alexa488-anti-mouse IgG. (E) Western blot showing RON5 levels in RON5_MYC_cKD parasites after 24, 48 and 72 hours of Atc treatment. All scale bars = 5 µm.

RONs 2/4/5/8 are the only rhoptry proteins known to localize to the moving junction and are believed to play an important role in host cell invasion. We have shown that the Coccidia-restricted RON8 is important but not absolutely required for invasion, suggesting that the remaining MJ RONs 2/4/5 compose an apicomplexan MJ core complex that constitutes the key invasion machinery employed across the phylum [25]. While the *Toxoplasma* genome encodes a RON4 paralog and two RON2 paralogs, RON5 appears to be a single copy gene with no isoforms [31]. Thus, we reasoned that disruption of RON5 was likely to yield unambiguous functional insight into the MJ core complex. Repeated attempts to disrupt *RON5* in the RHΔ*ku80* parasite strain were unsuccessful, further suggesting a critical role in parasite biology. To directly assess the function of RON5 using a conditional approach, we first generated a parasite strain containing a C-terminal 3xMYC epitope tag at the endogenous RON5 locus to improve detection of the protein and then replaced the endogenous RON5 promoter with a tetracycline-regulatable promoter element (TRE, which is composed of seven tandem TetO sequences immediately upstream of a constitutive, truncated SAG4 promoter, Figure 2A). To allow for various epitope tag combinations in downstream experiments, we similarly constructed a RON5-3xHA version of this strain (designated as RON5_MYC_cKD or RON5_HA_cKD). This second tagged conditional knockdown line also provided an independent confirmation of our results.

As expected from the truncated promoter contained within the TRE, parasites having undergone the desired recombination event show a lower level of RON5 expression compared to the parental line (Figure 2B, note also the upshift in migration of RON5C due to the presence of the epitope tag). Rhoptries are assembled *de novo* during each round of parasite division and protein traffic to the organelle is restricted to a narrow window during biosynthesis [32]. In agreement with this, we observe some mistargeting of RON5C under the control of the constitutive TRE promoter in the RON5cKD parasites (Figure 2C, -Atc). Similar mistargeting was previously observed when expression of other rhoptry proteins were placed under the control of the TRE and likely corresponds to protein synthesized outside of the rhoptry biosynthesis timeframe [33]. Importantly, a focus of RON5C signal is present in the rhoptry necks of each cell, as assessed by co-localization with RON11 (arrow, Figure 2C) and RON5C is clearly detectible in MJ rings during invasion (arrows, Figure 2D).

Treatment with anhydrotetracycline (Atc) to repress expression results in a steady loss of RON5 with protein levels dropping below detectability by 72 hours (Figure 2E). No gross effect on rhoptries was observed in parasites lacking RON5 as assessed by IFA with rhoptry body markers ROP2/3/4 (not shown) and the non-MJ rhoptry neck marker RON11 (Figure 2E, +Atc). Additionally, no defect in intracellular replication was detected in parasites lacking RON5 (data not shown).

### RON5 is critical for invasion and evacuole formation but not egress

**Figure 3:**
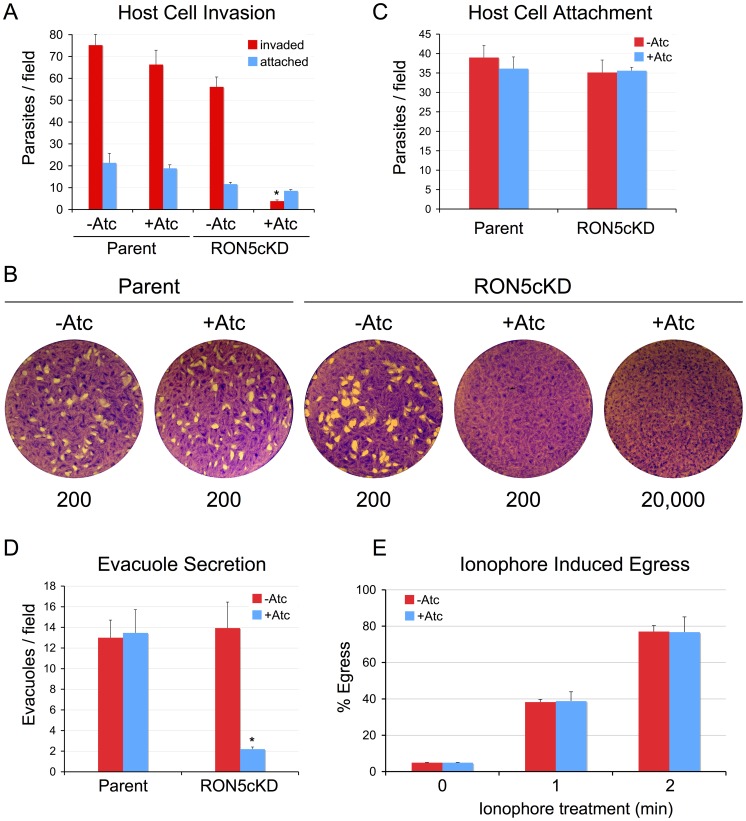
RON5 is critical for host invasion but not egress. (A–B) A major invasion defect is observed in parasites that lack RON5. (A) Parental or RON5_MYC_cKD parasites were grown for 72 hours −/+ Atc and then allowed to invade into fresh host cells for one hour. Following depletion of RON5, parasites show a nearly complete block in host penetration (asterisk, p-value <0.001). A corresponding increase in attached, uninvaded parasites is not observed (blue bars). A minor decrease in penetration is also seen for untreated RON5_MYC_cKD parasites, likely due to the lower levels of RON5 expressed in this strain relative to the parental line. (B) Parasites depleted of RON5 cannot form plaques in fibroblast monolayers. Parental or RON5_MYC_cKD parasites were grown 48 hours −/+ Atc and then infected into fresh fibroblast monolayers at a dose of 200 parasites per well and incubated for nine days. RON5_MYC_cKD parasites are unable to form plaques in the presence of Atc, even at an infective dose of 20,000 parasites per well. Numbers under images indicate infective dose. (C) Initial attachment is not affected by knockdown of RON5. Parasites were grown for 60 hours −/+ Atc before treatment with cytochalasin D to block motility and arrest the invasion process just after attachment. (D) Loss of RON5 eliminates secretion of rhoptry body proteins as assessed by evacuole formation (asterisk, p-value <0.001). Parasites were grown for 60 hours −/+ Atc before treatment with cytochalasin D to block invasion and allow evacuole formation. Evacuoles were detected by staining for ROP2/3/4. (E) Parasite egress is unaffected by loss of RON5. Parasites were grown 60 hours −/+ Atc and then induced to egress by treatment with calcium ionophore A23187 before fixation and staining for detection with anti-SAG1. No difference was seen in egress efficiency of parasites with or without RON5.

To test the importance of RON5 in host cell entry, we performed invasion assays on RON5cKD parasites depleted of RON5. In the absence of Atc treatment, a minor decrease in the invasive capacity of these parasites is observed relative to the parental line, likely corresponding to the lower levels of RON5 produced in the knockdown strain (Figure 3A, red bars). In contrast, a major block in invasion is observed following depletion of RON5 (Figure 3A, red bars), indicating that RON5 is critical for this process. The low level of invasion observed following Atc treatment may be the result of residual levels of RON5 present in some parasites or could indicate that parasites lacking RON5 are able to invade but only at very low levels. In an attempt to distinguish between these possibilities, we performed pulse invasion assays in order to observe Atc-treated RON5cKD parasites in the process of host cell penetration. While invasion events were rare in these assays, all penetrating parasites observed displayed detectable RON5 in the moving junction and/or rhoptry necks (Figure S2) suggesting invasion events in Atc-treated parasites are the result of residual RON5. We next performed plaque assays to better assess the invasion defect over the course of several lytic cycles. While wild-type parasites readily formed plaques in the presence or absence of drug treatment, no plaques were formed by the RON5cKD parasites in the presence of Atc, even with a hundred-fold higher parasite load (Figure 3B). Together, these results show the critical importance of RON5 for *Toxoplasma* invasion and suggest that RON5 may be essential for this process.

The block in parasite invasion in the absence of RON5 is not accompanied by a simultaneous increase in attached parasites (Figure 3A, blue bars). Thus, parasites depleted of RON5 either exhibit an attachment defect, or more likely, initially attach normally but then detach following a failure to invade as previously observed with disruption of RON8 and with knockdown of TgDHHC7 or TgARO [25], [33]. To distinguish between these two possibilities, we treated parasites with cytochalasin D to inhibit actin polymerization, arresting the invasion process just after apical reorientation but prior to penetration by disabling gliding motility [34]. Under these conditions, parasites depleted of RON5 were found to attach to host cells with the same efficiency as untreated or parental line parasites. This data indicates that initial attachment is not impaired and suggests that a failure to invade in the absence of RON5 is followed by gliding motility-based detachment and that these parasites are then washed away during invasion assay processing (Figure 3C).

During invasion, parasites inject a number of key effectors from the rhoptry body into the host cytosol to modulate host signaling and innate immunity [35]. Rhoptry secretion can be visualized independent of invasion by arresting the invasion process with cytochalasin D. Under these circumstances, an early stage MJ is still formed at the point of apical contact between the parasite and host cell surface and several rhoptry body proteins (ROPs) can be visualized entering the cell in membranous structures called evacuoles [36]. To determine the importance of RON5 for secretion of rhoptry body contents, we monitored the formation of evacuoles in parasites depleted of RON5. Although cytochalasin D-treated parasites lacking RON5 still attach normally (Figure 3C), a nearly complete loss of evacuole formation was observed indicating RON5 is critical for injection of rhoptry contents into the host cell (Figure 3D and Figure S1).

In addition to the roles in invasion and rhoptry secretion highlighted above, the MJ has also been suggested to play a role in host cell exit as RON4-positive MJ rings have been reported to form during egress [8], [11], [37]. To assess the importance of RON5 in this process, we induced egress using the calcium ionophore A23187. Under these conditions, we observed no defect in host cell exit as parasites with or without RON5 egressed with the same efficiency (Figure 3E). Collectively, these results demonstrate that RON5 plays a critical role in host cell invasion but is dispensable for egress. Our results with the RON5 knockdown are in agreement with the recent finding that ablation of rhoptry tethering (via knockdown of the rhoptry-localized palmitoyl acyl transferase TgDHHC7 and its putative substrate TgARO) similarly blocks invasion but not egress and highlights RON5 as a critical player in rhoptry mediated invasion [33], [38].

### The MJ RON core complex is disrupted in the absence of RON5

**Figure 4:**
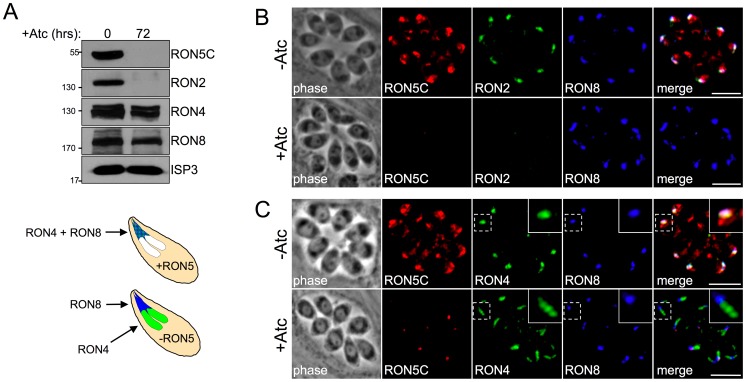
The MJ RON core complex is disrupted in the absence of RON5. (A) Western blot analysis of MJ RONs 2, 4 and 8 following RON5 knockdown. RONs 2, 4 and 8 were compared in the RON5_MYC_cKD strain with or without 72 hours of Atc treatment. RON2 levels are similarly reduced following knockdown of RON5. In contrast, only a minor decrease in RON4 and RON8 levels is observed. ISP3 serves as a loading control. (B) IFA showing RON2 is lost in RON5_HA_cKD parasites depleted of RON5 while RON8 levels and targeting are unaffected. Although a minor decrease in RON8 signal was observed by Western blot following depletion of RON5 (A), no change in RON8 was observed between vacuoles with or lacking RON5. Red: rat anti-HA antibody detected by Alexa594-anti-rat IgG. Green: rabbit anti-RON2 antibody detected by Alexa488-anti-rabbit IgG. Blue: mouse anti-RON8 antibody detected by Alexa350-anti-mouse IgG. (C) IFA showing RON4 is mistargeted to the rhoptry body in RON5_HA_cKD parasites the absence of RON5. RON4 normally co-localizes with RON5 and RON8 in the rhoptry neck. However, in parasites depleted of RON5, co-localization between RON4 and RON8 is lost with RON4 signal extended just posterior to RON8 indicating mistargeting to the rhoptry body. Red: rabbit anti-HA antibody detected by Alexa594-anti-rabbit IgG. Green: rat anti-RON4 antibody detected by Alexa488-anti-rat IgG. Blue: mouse anti-RON8 antibody detected by Alexa350-anti-mouse IgG. All scale bars = 5 µm.

To determine the impact of the loss of RON5 on the rest of the MJ complex, we examined the remaining MJ RON components by Western blot analysis. Interestingly, following RON5 knockdown, the RON2 signal is also eliminated, indicating that RON5 is critical for maintaining the stability of RON2 (Figure 4A). This complete loss of signal is specific to RON2 as the protein levels of RON4 and RON8 are not similarly impacted under these conditions. While RON2 protein levels closely mimic those of RON5 over a series of time points during RON5 knockdown, qPCR analysis showed no decrease in the transcription of RON2 (instead we surprisingly observe an approximately two-fold increase in RON2 transcripts), indicating that loss of RON2 occurs at the protein level (Figure S3). The dependence of RON2 upon RON5 was also clearly observed by IFA as parasites lacking RON5 also lack RON2 (Figure 4B).

In contrast to the destabilization of RON2, RON8 is intact and properly targeted to the rhoptry necks in the absence of RON5 (Figure 4B). While Western blot analysis of RON4 indicates that it is largely stable in the absence of RONs 5 and 2, IFA revealed a targeting defect with RON4 signal often observed throughout the rhoptry bodies but not in the necks (Figure 4C). While the interactions of individual RONs in the MJ complex are unknown, this loss of colocalization between RON4 and RON8 strongly suggests that these proteins do not directly interact in the absence of RONs 2 and 5. Collectively, these results demonstrate that RON5 is required for the stability of RON2 and proper targeting of RON4 and show that RON5 knockdown effectively constitutes a RON5/2 double knockdown.

### Establishment of a complementation system to probe RON5 function

**Figure 5:**
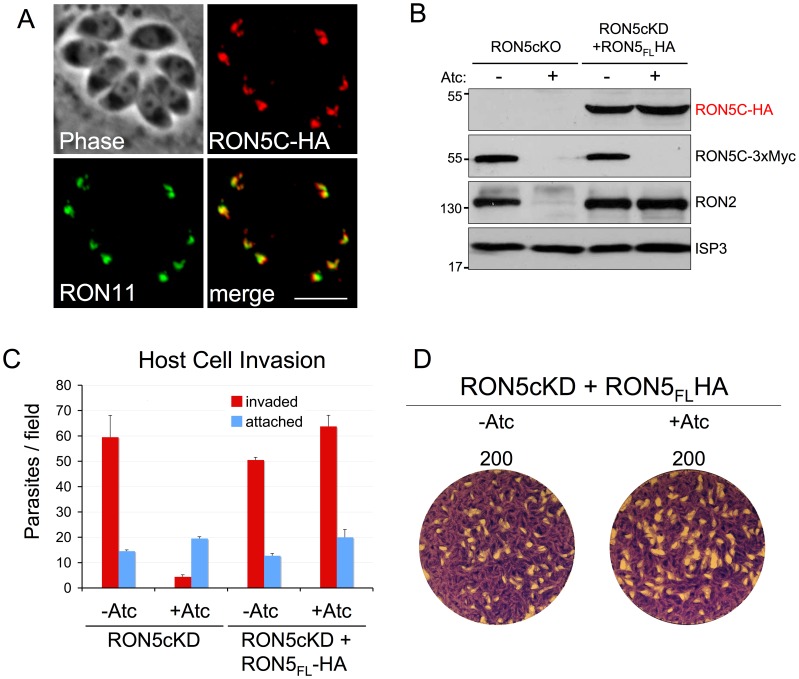
Establishment of a RON5cKD functional complementation system. (A) IFA showing rhoptry neck targeting of a second copy of RON5 with a C-terminal HA tag. RON5-HA targets properly to the rhoptry neck, as assessed by co-localization with RON11. Red: rabbit anti-HA antibody detected by Alexa594-anti-rabbit IgG. Green: rat anti-RON11 antibody detected by Alexa488-anti-rat IgG. Scale bar = 5 µm. (B) Western blot showing rescue of RON2 stability in Atc treated RON5_MYC_cKD by stable expression of a second copy of RON5. Expression of an HA tagged second copy of *RON5* is unaffected by Atc treatment while expression of the endogenous, MYC-tagged copy of *RON5* under the control of the tet-regulatable promoter is eliminated. While repression of endogenous *RON5* results in degradation of RON2, expression of the complementing *RON5* second copy fully rescues RON2 stability. ISP3 serves as a loading control. A complete rescue of (C) invasion and (D) restored plaque formation is also observed in RON5_MYC_cKD parasites complemented with a second copy of RON5.

The effect of RON5 knockdown on the integrity of the MJ complex and on invasion raises the question as to what regions of RON5 are necessary for maintaining stability of RON2 as well as whether any RON5-specific roles during invasion exist. To explore these questions, we established a functional complementation system in our RON5_MYC_cKD strain by targeting a full-length RON5 expression cassette under the control of its endogenous promoter to the UPRT locus. To distinguish this copy of RON5 from the MYC-tagged, regulatable copy transcribed from the endogenous locus, we engineered an HA epitope tag at the C-terminus. As expected, this HA-tagged version of RON5 targets properly to the rhoptry necks, co-localizing with RON11 (Figure 5A). Expression of this second copy of RON5, which is insensitive to Atc as it is driven from the RON5 promoter, fully rescues the stability of RON2 upon knockdown of endogenous RON5 (Figure 5B). In addition, complementation with full-length RON5 rescues invasion to wild-type levels and restores the ability of these parasites to plaque in the presence of Atc (Figure 5C–D).

### RON5N/C processing is dispensable for MJ function

**Figure 6:**
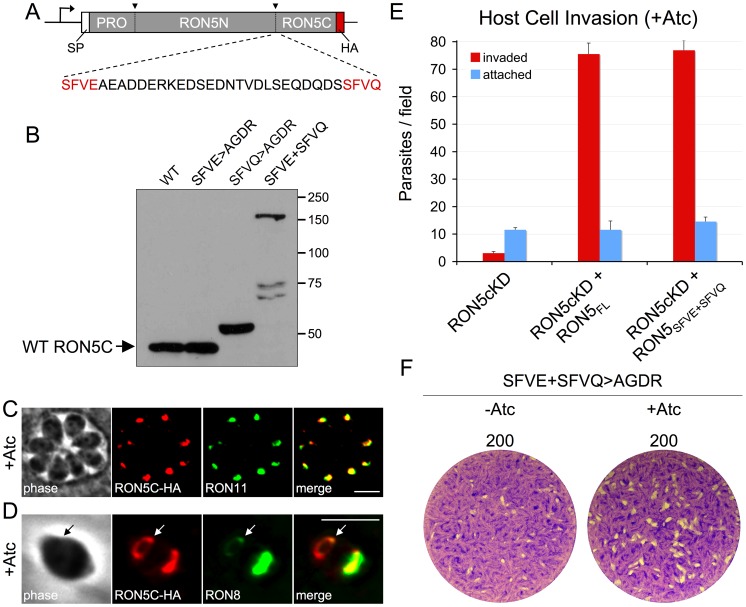
RON5N/C processing is dispensable for MJ complex function. (A) Diagram showing location and sequence context of the two putative RON5N/C processing sites SFVE (residues 1258–1262) and SFVQ (residues 1288–1291). To assess the importance of these sites for RON5N/C processing, a second copy of RON5 with a C-terminal HA tag and harboring various mutations of these sites was expressed in parasites. (B) Western blot showing SDS-PAGE migration of RON5N/C processing mutants from lysates of freshly egressed, extracellular parasites. Migration of RON5C in the indicated mutants was compared with wild-type RON5C (also a second copy with a C-terminal HA tag). The SFVE>AGDR mutation has no effect on migration of RON5C. The SFVQ>AGDR mutation results in a small upshift in RON5C migration consistent with a shift in processing to an upstream site. In contrast, the SFVE>AGDR+SFVQ>AGDR double mutant shows a major upshift, indicating a block in RON5N/C processing. The lower, faint bands in the double mutant lane are likely breakdown products. IFA of intracellular (C) or invading (D) RON5_MYC_cKD parasites complemented with RON5N/C processing mutant and grown for 72 hours with Atc to knockdown endogenous RON5. (C) Trafficking to the rhoptry neck is unchanged in the RON5N/C processing mutant as shown by colocalization with RON11. Red: rabbit anti-HA antibody detected by Alexa594-anti-rabbit IgG. Green: rat anti-RON11 antibody detected by Alexa488-anti-rat IgG. (D) The RON5N/C processing mutant is seen in the MJ (arrows) during invasion where it colocalizes with RON8. Red: rabbit anti-HA antibody detected by Alexa594-anti-rabbit IgG. Green: mouse anti-RON8 antibody detected by Alexa488-anti-mouse IgG. All scale bars = 5 µm. Complementation of RON5_MYC_cKD with the RON5N/C processing mutant completely rescues (E) invasion and (F) restores plaque formation following Atc treatment.

We next employed this system to assess the importance of processing of RON5 into RON5N and RON5C. To determine the site of processing, we scanned the RON5 sequence to identify candidate sites that match the consensus P1–P4 sequence characterized in other rhoptry protein processing events (SΦXE, where Φ is a hydrophobic residue and X is any residue) [39]. A single match was identified (SFVE, residues 1258–1262) within the region where processing is expected to occur based on SDS-PAGE migration of the mature N- and C-terminal fragments and peptide coverage generated from mass spectrometric analysis of RON5N and RON5C (Figure 6A) [11], [13]. However, mutagenesis of all four residues of this site (SFVE>AGDR, expected to completely block processing) in a second copy of RON5 did not affect migration of the C-terminal fragment by SDS-PAGE relative to the wild-type protein, demonstrating that this mutant was still processed (Figure 6B, SFVE>AGDR).

We have recently shown that processing of the rhoptry protein TLN1 occurs at a similar sequence containing a glutamine instead of aspartic acid (SFVQ) [28]. An SFVQ site is also present within the region where RON5 N/C processing is expected to occur (residues 1288–1291, Figure 6A). Similar mutagenesis of this site (SFVQ>AGDR) results in a modest upshift of RON5C that does not agree with a block in processing to separate RON5N and C, but is consistent with processing upstream at the SFVE site (Figure 6B, SFVQ>AGDR). To test if this was the case, we generated a double mutant at both sites and observe a large upshift in this mutant to the approximate size expected for uncleaved RON5N/C minus its N-terminal pro domain, indicating a block in RON5N/C processing (Figure 6B, SFVE+SFVQ). These results indicate either that processing of RON5 is favored at SFVQ and shifted to SFVE upon ablation of this site, or that processing occurs at both sites in the endogenous protein.

To assess the functional impact of the failure to separate RON5N/C, we complemented the RON5_MYC_cKD strain with the double processing mutant. Despite the block in RON5N/C processing, this mutant was found to target to the rhoptry necks and MJ ring in an indistinguishable manner from the wild-type protein. This was also true following Atc depletion of endogenous RON5, ruling out the possibility that endogenous RON5 supports proper trafficking of heterogeneous complexes containing both processed and unprocessed forms of RON5 (Figure 6C–D). Surprisingly, the processing mutant also fully rescued the stability of RON2 (not shown), invasion and the ability to form plaques upon depletion of endogenous RON5 (Figure 6E–F). Collectively, these results demonstrate that proteolytic separation of RON5N and RON5C is not important for MJ complex integrity, trafficking or function, a surprising result considering that multiple processing sites are maintained within this region of the protein.

### RON5C is required to stabilize RON2

**Figure 7:**
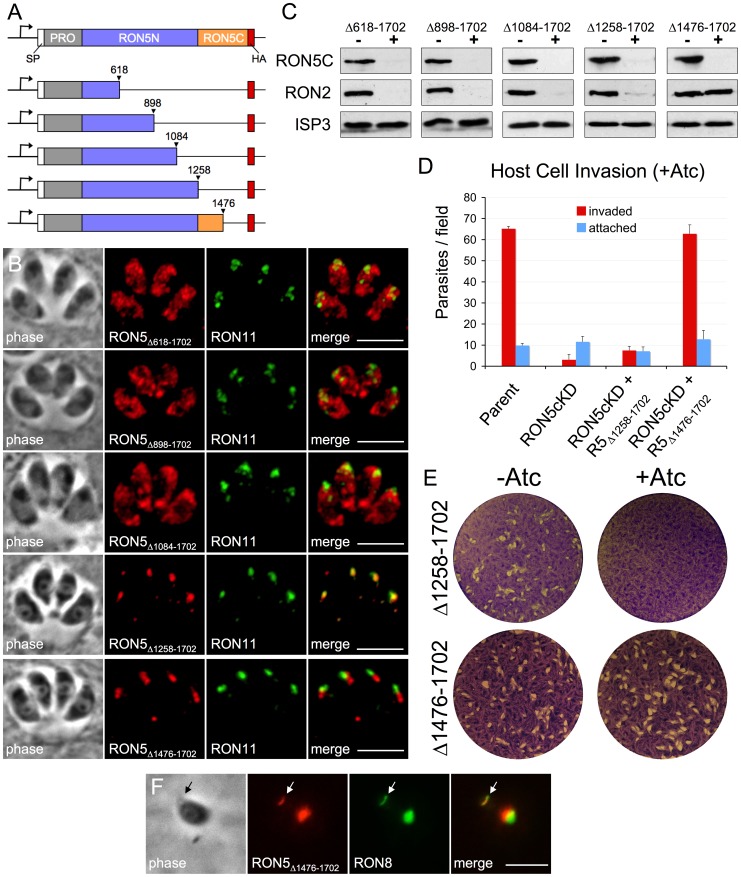
RON5C is required to stabilize RON2. (A) Diagram showing design of a series of C-terminal truncations of RON5. Truncation mutants each containing a C-terminal HA tag and were expressed from the UPRT locus. (B) IFA showing localization of indicated RON5 truncation mutants. Gross mislocalization is seen for the Δ618-1702, Δ898-1702 and Δ1084-1702 truncations. In contrast, the Δ1258-1702 and Δ1476-1702 truncations were found to target to the rhoptry necks as assessed by co-localization RON11. A slightly more posterior localization relative to RON11 was observed for RON5_Δ1476-1702_. Red: rabbit anti-HA antibody detected by Alexa594-anti-rabbit IgG. Green: rat anti-RON11 antibody detected by Alexa488-anti-rat IgG. All scale bars = 5 µm. (C) Western blot assessing the ability of various RON5 truncation mutants to rescue stability of RON2 in the absence of endogenous RON5. RON2 levels were compared following 72 hours of Atc treatment in RON5_MYC_cKD parasites complemented with a series of RON5 truncation mutants. Endogenous RON5C levels were monitored with an anti-MYC antibody to ensure depletion of endogenous RON5. Complete degradation of RON2 is still observed in cells complemented with the Δ618-1702, Δ898-1702, Δ1084-1702 and Δ1258-1702 RON5 truncation mutants. In contrast, complementation with RON5_Δ1476-1702_ completely rescues the stability of RON2. ISP3 serves as a loading control. (D–E) Complementation with RON5_Δ1258-1702_ fails to rescue (D) invasion or (E) plaque formation in the absence of endogenous RON5 while RON_Δ1476-1702_ provides a complete rescue of invasion and plaque formation. (F) IFA showing localization of RON5_Δ1476-1702_ in an invading parasite after treatment with Atc for 72 hours to knockdown endogenous RON5. The truncated RON5_Δ1476-1702_ protein can be seen in the MJ (arrow) where it colocalizes with RON8. Red: rabbit anti-HA antibody detected by Alexa594-anti-rabbit IgG. Green: mouse anti-RON8 antibody detected by Alexa488-anti-mouse IgG. Scale bar = 5 µm.

Since RON5N/C processing is not required for function, we tested the possibility that RON5C is dispensable all together. To guide the design of addition mutants, we generated an alignment between *Toxoplasma* and *P. falciparum* RON5 sequences to determine conservation hot spots that might encode key regions for interaction with other complex members and function (Figure S4). The alignment reveals three general regions of varying conservation between the two species, the highest of these being the C-terminal half of RON5N (residues 897–1257). An intermediate level of conservation is seen for the N-terminal half of RON5N (residues 315–896) while the lowest level of conservation is observed in an area that roughly corresponds to *Toxoplasma* RON5C (residues 1258–1702). Using this information together with secondary structure prediction, we designed a series of C-terminal truncations and expressed each of these mutants from the UPRT locus in the RON5_MYC_cKD strain (Figure 7A). Three of these truncations (Δ618-1702, Δ898-1702, and Δ1084-1702), each of which removes the entire RON5C region as well as portions of RON5N, were found to grossly mistarget (Figure 7B). For each of these mutants the mistargeted signal was sometimes absent in individual parasites within a clonal line, suggesting cell cycle variance. Indeed, co-staining for the IMC apical cap marker ISP1 showed that HA signal was only observed in parasites in the process of assembling daughter buds (and thus new rhoptries), indicating that these RON5 mutants are likely degraded following a failure to target to the rhoptry neck as previously observed for other RON targeting mutants (Figure S5A) [40].

In contrast, truncations which remove half (Δ1476-1702) or all (Δ1258-1702) of RON5C continue to target to the rhoptry necks (Figure 7B), although some cell-cycle dependent mistargeting was still observed (not shown). Interestingly, the majority of RON5_Δ1476-1702_ signal localized slightly posterior to non-MJ complex markers for the rhoptry neck, although the significance of this slight shift in localization is unclear. These results indicate that RON5C is dispensable for trafficking while the C-terminal region of RON5N is necessary for localization to the rhoptry necks.

To further explore trafficking determinants, we targeted proRON5 for deletion. Although the site(s) of proRON5 cleavage is not known, a candidate SFVE is found at residues 311–314, which agrees with the N-terminal boundary of RON5N suggested by previous proteomic analyses [11], [12]. To determine if proRON5 is important for RON5 targeting, we created an inframe deletion removing the region between the signal peptide and putative pro cleavage site (residues 36 to 314) in the HA/FLAG double-tagged RON5 expression cassette. Detection with both HA (Figure S5B) and FLAG (data not shown) epitopes showed gross mistargeting of this protein. While this result demonstrates that proRON5 is necessary for RON5 targeting, our C-terminal truncation analysis indicates that proRON5 is not sufficient for this process. Together, these results suggest that the pro region as well as the C-terminal portion of RON5N play a role in proper targeting, possibly through ensuring proper RON5 folding and/or facilitating interaction with other members of the MJ RON complex.

We next evaluated the ability of our C-terminal truncation mutants to rescue the stability of RON2 upon knockdown of endogenous RON5. As expected, mutants which failed to traffic to the rhoptry neck (Δ618-1702, Δ898-1702 and Δ1084-1702) also failed to stabilize RON2 in the absence of endogenous RON5 (Figure 7C). While the Δ1258-1702 truncation mutant lacking RON5C does target to the rhoptry neck, it also fails to rescue RON2 stability, demonstrating that although RON5N/C processing is dispensable, RON5C is required for RON2 integrity. In contrast, the Δ1476-1702 truncation lacking the C-terminal 227 residues of RON5C completely rescues RON2 stability (Figure 7C). To monitor both the impact on penetration and downstream intracellular survival, we performed invasion and plaque assays using the RON5_MYC_cKD strain complemented with the Δ1258-1702 or ΔA1476-1702 mutants. We found that the Δ1258-1702 mutant was unable to rescue invasion or form plaques upon knockdown of endogenous RON5 while the Δ1476-1702 mutant restored both of these phenotypes (Figure 7D–E). As expected, RON5_Δ1476-1702_ localized to the MJ of invading parasites following knockdown of endogenous RON5 (Figure 7F). Taken together with our analysis of N/C processing, these results identify residues 1292–1475 of RON5 as critical for maintaining RON2 stability and suggest this domain may directly interact with RON2.

## Discussion

The establishment of a tight-junction interface between invading apicomplexan parasites and their host cells was first observed by electron microscopy over 30 years ago [41]. More recently, the exciting discovery that a complex of rhoptry neck proteins is secreted into this tight-junction provided candidates for understanding the molecular basis for this unique mechanism of host cell penetration [11]. While a relatively thorough characterization of RON protein topology within the MJ has been carried out, a hydrophobic stretch of residues in the N-terminus of RON5 has been noted as a potential transmembrane region, which would impact the positioning of RON5 in this model (see [14], [29]). We show here that the RON5 N-terminal domain in which this hydrophobic region is contained is not a part of the mature MJ complex. Instead, this pro domain likely plays roles in RON5 folding or trafficking as deletion of proRON5 resulted in gross mistargeting of the remainder of the protein. While the RON5 pro region is necessary for trafficking, it does not appear to be sufficient as C-terminal truncations of RON5N also result in mistargeting. The C-terminal region of RON5N is the most highly conserved portion of the protein, potentially suggesting that this region is critical for complex assembly in addition to trafficking. Importantly, a version of RON5 lacking the entire RON5C domain (RON5Δ1258-1702) targets to the rhoptry necks but cannot rescue RON2 stability (see below), showing that RON5 contains the necessary rhoptry neck targeting information independent of RON2.

Knockdown of RON5 demonstrates a critical importance in organizing the MJ RON complex. The specific impact on RONs 2 and 4 following depletion of RON5 provides experimental support for the idea that RONs 2/4/5 constitute a MJ core complex, consistent with their conservation across the phylum. In contrast, RON8 appears to represent a coccidial innovation that contains its own targeting information to facilitate sorting to the rhoptry necks. The simultaneous loss of RON2 upon RON5 knockdown may be due to Endoplasmic Reticulum-Associated Degradation (ERAD) quality control systems that sense misfolded proteins, extract them from the ER and target them to the proteasome [42], [43]. This specific degradation of RON2 but not other MJ complex components in the absence of RON5 suggests that RON5 may directly bind RON2 (although this could also be achieved indirectly through other complex components) and ensure its proper folding or mask a RON2-encoded signal for ER retention and degradation similar to characterized protein complexes in other systems [42].

In contrast to RON2, the soluble MJ component RON4 is not degraded but fails to target to the rhoptry necks in the absence of RON2/5, indicating that RON4 contains targeting information to enter the rhoptries, but requires interaction with RON2/5 to ultimately reach the necks of the organelle. Little is known about the determinants for sub-domain trafficking within the rhoptries. Interestingly, a reverse scenario was observed for the *Toxoplasma* rhoptry body protein ROP1 and the *P. falciparum* rhoptry body protein RAP1, each of which mistarget to the rhoptry neck following truncation of C-terminal residues [26], [44].

Loss of RON2 and mistargeting of RON4 following RON5 knockdown indicates that RON5 serves an escorter role that is required for MJ core complex trafficking and integrity. A somewhat similar scenario was reported for a recently identified RON complex in *Toxoplasma* consisting of RON9 (a predicted transmembrane protein) and RON10 (a predicted soluble protein), although in this case both partners are required as gross mistargeting (but not degradation) and total loss of rhoptry localization of each protein occurs in the absence of the other [40]. Thus, the stability and trafficking of protein complexes targeted to the rhoptry neck appears to be commonly achieved through the presence of escorters, as has been observed for certain micronemal proteins [45], [46].

Blocking proteolytic separation of RON5 N and C requires mutation of two sites within RON5, suggesting there is some selective pressure to maintain this processing event. Despite this fact and to our surprise, we find that N/C processing during maturation is not important for RON5 functions in MJ complex organization or parasite invasion. Therefore, while RON5C is necessary for RON2 stability, processing is not required for some structural rearrangement of these domains as one might infer from the presence of multiple sites at which N/C processing can occur. While a number of processing events have been characterized in rhoptry proteins and the suspected maturase TgSUB2 is thought to be essential, the known functional importance of processing is limited to the removal of N-terminal pro-domains which are involved in trafficking and no longer needed upon reaching their destination [26], [28], [47]. While the key importance of RON5 for invasion seemed to provide an excellent opportunity for determining the role of rhoptry protein processing beyond such trafficking functions, the lack of any effect on invasion when separation of RON5N/C is blocked suggests that cleavage may play an extremely subtle role in MJ complex function or that parasites can rapidly adapt when processing is blocked.

Previously, peptides that interfere with the interaction of RON2 and AMA1 were found to block invasion but not evacuole formation [19]. Interestingly, we find that knockdown of RON5 (and RON2) results in a marked decrease in evacuole formation. This indicates a critical role for the MJ RON complex in facilitating rhoptry secretion and suggests that rhoptry secretion proceeds in a stepwise fashion with deployment of the MJ RONs from the rhoptry neck occurring prior to secretion of rhoptry body contents.

The fact that RON5 is present in the MJ suggests that in addition to its importance as an escorter ensuring stability and proper targeting of RONs 2 and 4, RON5 may also serve direct roles in host cell penetration. Indeed, parasites that have been depleted of RON5 fail to establish an observable MJ and the rare penetration events that are observed appear to be supported by residual RON5 in these individual cells. However, at this point we cannot distinguish between invasion defects resulting directly from loss of RON5, indirectly from loss of RON2, or both. Furthermore, mistargeting of RON4 suggests that RON4-specific functions are likely also impaired in these conditions. Future work aimed at characterizing the potential direct interaction between RON5 and RON2 may provide insight to design new RON5 mutants that can stabilize RON2 and allow investigation of any RON5-specific roles in invasion. Additionally, RON2-specific knockdowns are needed that will allow RON2 function to be directly probed at the genetic level. In conclusion, our results highlight the importance of the MJ RON core complex for *Toxoplasma* invasion. These results are particularly significant given the recent finding that AMA1 disruption impacts adhesion but not penetration [24]. Taken together with the importance of RON4 for invasion by *P. berghei* sporozoites, these findings indicate a key role for the RON 2/4/5 complex in establishing the apicomplexan moving junction and facilitating host penetration [23].

## Materials and Methods

### Ethics statement

Antibodies were raised in rats under the guidelines of the Animal Welfare Act and the PHS Policy on Humane Care and Use of Laboratory Animals. Specific details of our protocol were approved by the UCLA Institutional Animal Care and Use Committee, known as the Chancellor's Animal Research Committee (protocol # 2004-055-31C).

### *Toxoplasma* and host cell culture

*T. gondii* RHΔ*hpt* (parental) strain and modified strains were maintained in confluent monolayers of human foreskin fibroblast (HFF) host cells as previously described [48].

### Antibodies

The following *Toxoplasma* primary antibodies were used in IFA or Western blot: mouse polyclonal anti-RON5C [13], polyclonal rat anti-RON11 [33], rabbit anti-RON2 [25], rat polyclonal anti-RON4 (see below), rabbit anti-RON4 [11], mouse polyclonal anti-RON8 [13], anti-ROP2/3/4 mAb TA7 1A11 [49], rabbit anti-ROP13 [50], rabbit anti-SAG1 [51], anti-ISP1 mAb 7E8 [52], mouse polyclonal anti-ISP3 [52], monoclonal mouse anti-F1-ATPase beta subunit 5F4 (Bradley, unpublished), rabbit anti-proROP4 UVT70 [30]. Hemagglutinin (HA) epitope tags were detected with mouse mAb HA.11 (Covance), rabbit polyclonal anti-HA (Invitrogen) or rat mAb 3F10 (Roche). MYC epitope tags were detected with mouse mAb 9E10 (Neomarkers). FLAG epitope tags were detected with mouse anti-FLAG mAb M2 (Sigma). For generation of rat anti-RON4 sera, a portion of the RON4 coding sequence comprising residues 85–983 was recombinantly expressed in *E. coli* BL-21DE3 cells and purified over Ni-NTA agarose (Qiagen) as previously described [53]. The resulting protein was injected into a rat for anti-sera production.

### Western blots

Freshly lysed parasites were collected for Western blots. In time course analysis of protein levels, time points were designed to correspond with monolayer lysis. All parasite samples were counted on a hemocytometer to ensure equivalent loading between lanes.

### Light microscopy and image processing

Fixation and immunofluorescence staining of *T. gondii* were carried out as previously described [52]. Image stacks were collected at z-increments of 0.2 µm with an AxioCam MRm CCD camera and AxioVision software on an Axio Imager.Z1 microscope (Zeiss) using a 100× oil immersion objective. Deconvolved images were generated using manufacturer specified point-spread functions and displayed as maximum intensity projections.

### Generation of RON5 endogenous epitope tags

The endogenous tagging vector p3xHA.LIC.DHFR [54] was first modified to replace the DHFR selectable marker cassette with a chloramphenicol acetyl-transferase (CAT) selectable marker between the restriction sites *HindIII*/*XbaI* resulting in the plasmid p3xHA.LIC.CAT. A portion of the genomic locus of RON5 up to but not including the stop codon was PCR amplified from *Toxoplasma* genomic DNA (primers P1/P2, Table S1) and inserted into p3xHA.LIC.CAT or p3xMYC.LIC.CAT by ligation-independent cloning [55] to generate the vectors pRON5-3xHA.LIC.CAT and pRON5-3xMYC.LIC.CAT. These plasmids were linearized with *PstI* and transfected into the TATiΔ*ku80* parasite line [56]. Following selection with chloramphenicol, parasites were cloned by limiting dilution and a clone expressing the tagged protein of interest was isolated and designated RON5-3xHA or RON5-3xMYC.

### Generation and complementation of RON5cKD parasites

For direct replacement of the RON5 promoter with the conditional TetOSAG4 promoter by homologous recombination, 5′ (primers P3/P4) and 3′ (primers P5/P6) regions flanking the RON5 promoter were PCR amplified from *Toxoplasma* genomic DNA and cloned into the vector pDT7S4myc [56] between *NdeI* and *BglII*/*AvrII* sites, respectively. The resulting vector, pTS4-RON5-DHFR, was linearized with *ApaI* and transfected into RON5-3xMYC or RON5-3xHA parasites. Following selection with 1 µM pyrimethamine, parasites were cloned by limiting dilution and genomic DNA from individual clones was analyzed for RON5 promoter replacement (primers P7/P8). A clone that had undergone the intended recombination event was designated RON5_MYC_cKD or RON5_HA_cKD.

For expression of a complementing second copy of RON5, the RON5 promoter was PCR amplified from *Toxoplasma* genomic DNA (primers P9/P10) and inserted into the UPRT targeting vector pUPRTKO-HA [57] between *SpeI* and *BamHI* by blunting both the digested vector and PCR amplicons, resulting in the vector pUPRTKO-RON5-promoter-HA. The full length RON5 coding sequence was PCR amplified from a *Toxoplasma* cDNA library (primers P11/P12) and inserted into this vector between *BglII*/*NotI* sites to generate the vector pUPRTKO-RON5-HA. This vector was linearized with *NsiI* and transfected into RON5_MYC_cKD parasites followed by selection with 5 µg/ml 5-fluorodeoxyuridine to facilitate targeted replacement of the UPRT locus [58].

### Generation RON5 mutants and double epitope tagged versions of RON5

For site directed mutagenesis, a portion of the RON5 coding sequence between the restriction sites *SmaI* and *NotI* was digested from the vector pUPRTKO-RON5-HA and inserted into the cloning vector pJet1.2 (Fermentas). Site-directed mutants were generated by Quick Change Mutagenesis (Stratagene) with mutagenesis primers as follows (forward primer given, reverse complement was also used): SFVE>AGDR (P13) and SFVQ>AGDR (P14).

For expression of double tagged RON5 to monitor proRON5, a FLAG epitope tag version of the vector pUPRTKO-RON5-HA was first generated by PCR amplifying the 3′ UTR with a forward primer encoding the FLAG epitope sequence (primers P15/P16) and inserting this amplicon between *NotI*/*EcoRV*, replacing the inframe fusion to a C-terminal HA tag with a FLAG tag (pUPRTKO-RON5-FLAG). A portion of the 5′ RON5 coding sequence was PCR amplified (primers P11/P17) and inserted into the cloning vector pJet1.2 (Fermentas). An HA epitope was then inserted into the RON5 coding sequence between residues 35 and 36 using Quick Change Mutagenesis (P18) and this modified coding sequence was inserted into the vector pUPRTKO-RON5-FLAG between *BglII/RsrII* resulting in the vector pUPRTKO-RON5-PRO-HA-C-FLAG.

For generation of an inframe deletion of proRON5, a portion of the RON5 promoter and 5′ coding sequence was PCR amplified from the vector pUPRTKO-RON5-PRO-HA-C-FLAG with a reverse primer encoding a *KasI* site (primer P19/P20) and re-inserted into this vector between *NheI/AscI*. A portion of the RON5 coding sequence was then PCR amplified (primers P21/P22) and inserted between *KasI/AscI*, resulting in the vector pUPRTKO-RON5Δpro-N-HA-C-FLAG. For generation of C-terminal truncations of RON5, truncated portions of the RON5 coding sequence were amplified (Δ618-1702: P11/P23; Δ898-1702: P11/P24; Δ1019-1702: P11/P25; Δ1258-1702: P11/P26; Δ1476-1702: P11/P27) and inserted into the vector pUPRTKO-RON5-promoter-HA between *BglII/NotI* to generate the indicated C-terminal truncations.

### Plaque assays

Parasites were grown 48 hrs −/+1.5 µg/ml Atc, syringe lysed and infected into 6-well dishes containing fresh, confluent HFF monolayers −/+ Atc. Cultures were allowed to grow nine days before fixation with methanol followed by staining with crystal violet.

### Invasion, evacuole and egress assays

Invasion assays were performed as previously described [59]. Briefly, parasites were grown 72 hrs −/+1.5 µg/ml Atc, monolayers were washed with PBS and intracellular parasites were collected by scraping and passage through a 27-gauge needle. Equivalent parasite numbers were resuspended in pre-warmed media and allowed to infect HFF monolayers on coverslips for one hour. Monolayers were then washed, fixed with EM-grade 3.7% formaldehyde/PBS (Biosciences, Inc.), blocked with PBS/3% BSA for 30 min and incubated with rabbit anti-SAG1 diluted in PBS/3%BSA for 1 hr. After washing, samples were permeabilized in PBS/3% BSA/0.1% Triton X-100 for 30 min and then incubated with mAb 5F4 diluted in PBS/3% BSA for one hour. Following incubation with secondary antibodies, samples were examined by fluorescence microscopy and parasites were scored as invaded (SAG1−, 5F4+) or attached (SAG1+, 5F4+). Invasion assays were performed in triplicate, five fields were counted on each replicate coverslip and the average number of invaded and attached parasites per field was calculated. Synchronized pulse invasion assays were performed as previously described with parasites that had been pre-treated 72 hrs −/+ Atc [60].

For evacuole assays, parasites were grown −/+1.5 µg/ml Atc for 24 hours, then infected into fresh HFF monolayers and allowed to grow an additional 36 hours −/+1.5 µg/ml Atc to allow large vacuoles to form. Intracellular parasites were collected by scraping and passage through a 27-gauge needle. Evacuole assays were then performed as previously described [61]. The number of evacuoles was counted blind across five fields per coverslip on three independent coverslips per sample and the average number per field was calculated.

Egress assays were performed as previously described [62]. Briefly, parasites were grown −/+1.5 µg/ml Atc for 24 hours, then infected into fresh HFF monolayers on coverslips and allowed to grow an additional 36 hours −/+1.5 µg/ml Atc. Coverslips were then washed with PBS and incubated in 1 µM calcium ionophore A23187 (Sigma) diluted in Hank's Balances Salts Solution at 37°C before being fixed in methanol and processed for IFA with rabbit anti-SAG1. At least 100 vacuoles per coverslip were counted across five fields on three independent coverslips per sample and scored as egressed or not egressed. For each of the above assays, experiments were repeated at least twice and values from a representative experiment are shown as the mean ± SD.

### Quantitative real time PCR

RON5_HA_cKD parasites were grown 72 hrs −/+1.5 µg/ml Atc. Total parasite RNA was harvested with TRiZol (Invitrogen), purified using a RNaEASY column (Qiagen) and used as to generate cDNA with the iSCRIPT kit (BioRad). Relative amounts of RON5 (P28/P29) and RON2 (P30/P31) mRNA were quantified by qPCR using iQ Sybr Green (kapaBiosystems) and normalized to actin (P32/P33) using ΔΔCt statistical analysis [63].

## Supporting Information

Figure S1 **RON5 is critical for evacuole formation.** IFA visualization of evacuole production by RON5cKD parasites. Representative images are shown for parasites with or without Atc treatment and individual evacuole trails are indicated (arrows). A dramatic decrease in evacuoles is seen following depletion of RON5. Results are quantified in Figure 3D. Green: mouse anti-ROP2/3/4 antibody detected by Alexa488-anti-mouse IgG.
(TIF)

Figure S2 **Rare invading RON5cKD parasites following Atc treatment always show visible levels of RON5.** IFA showing a representative example of an invasion event by a RON5cKD parasite following 72 hours of Atc treatment. Note that while RON8 is robustly detected in the rhoptry necks, RON5 is barely detectible in the rhoptry neck (although clearly detected in the MJ - compare with untreated invading parasite shown in Figure 2D). Such invasion events were rare and were always accompanied by visible levels of RON5 in the MJ (arrows) and/or rhoptry necks. We visualized >100 invasion events across multiple experiments that all scored positive for RON5. This indicates that these rare invasion events are the result of residual RON5 that persists even after several days of treatment with Atc. Red: rabbit anti-HA antibody detected by Alexa594-anti-rabbit IgG. Green: mouse anti-RON8 antibody detected by Alexa488-anti-mouse IgG. Scale bar = 5 µm.
(TIF)

Figure S3 **RON2 levels closely mimic RON5 levels during RON5 knockdown and destabilization of RON2 occurs at the protein level.** (A) Western blot showing RON2 and RON5C levels after 24, 48 and 72 hours of Atc treatment. RON2 levels closely mimic diminishing RON5C levels showing that RON2 stability is dependent upon RON5. (B) qPCR analysis of RON5 and RON2 mRNA levels normalized to actin following 72 hours with or without Atc treatment. While a 19-fold decrease in RON5 transcripts is observed after Atc treatment, RON2 mRNA levels are not decreased and in fact show a small (∼2-fold) but reproducible increase. Data are representative of two independent experiments.
(TIF)

Figure S4 **Alignment of RON5 orthologs.** Alignment showing the level of conservation of RON5 sequence features between *Toxoplasma* and *P. falciparum* (GenBank accession numbers ACY08774 and ADV19051, respectively). Three general regions of differential conservation are seen: the highest level of conservation corresponds with the C-terminal portion of TgRON5N (residues 897–1257, red line) while the N-terminal portion of TgRON5N (residues 315–896, blue line) corresponds with a region of middle level conservation and TgRON5C (residues 1258–1702, green line) corresponds with a region of low conservation. The most N-terminal portion of TgRON5N as well as proTgRON5 show very low conservation with PfRON5. Alignment was generated using ClustalX and displayed using BoxShade (http://mobyle.pasteur.fr/cgi-bin/portal.py?#forms::boxshade).
(TIF)

Figure S5 **Analysis of mistargeted RON5 mutants by IFA.** (A) Cell cycle variance of RON5 truncation mutant signal. RON5_Δ1084-1702_ was detected in cells in the process of assembling daughter parasites (upper vacuole, note ISP1 labeling of two daughter IMC apical caps within each parasite in addition to the maternal apical cap signal) but not in non-dividing cells (lower vacuole, only maternal IMC apical cap signal is seen). The same phenomenon was also seen for other C-terminal truncation mutants (data not shown). Red: rabbit anti-HA antibody detected by Alexa594-anti-rabbit IgG. Green: anti-ISP1 mAb 7E8 detected by Alexa488-anti-mouse IgG. Blue: rat anti-RON11 antibody detected by Alexa350-anti-rat IgG. All scale bars = 5 µm. (B) Inframe deletion of proRON5 results in a failure to target to the rhoptry neck with signal accumulating in a region posterior to the rhoptry bodies, likely corresponding with the parasite Golgi. Gross mistargeting was also seen with staining for FLAG (data not shown). Red: mouse anti-HA antibody detected by Alexa594-anti-mouse IgG. Green: rabbit anti-ROP13 antibody detected by Alexa488-anti-rabbit IgG.
(TIF)

Table S1 **Primers used in this study as discussed in text.** Restriction sites and mutated or inserted bases are shown in lowercase.
(TIF)
