# Supplementary figures and images for: RON5 Is Critical for Organization and Function of the Toxoplasma Moving Junction Complex
Source: PLoS Pathog. 2014 Mar 20;10(3):e1004025. doi: 10.1371/journal.ppat.1004025 (PMC3961375; doi:10.1371/journal.ppat.1004025)

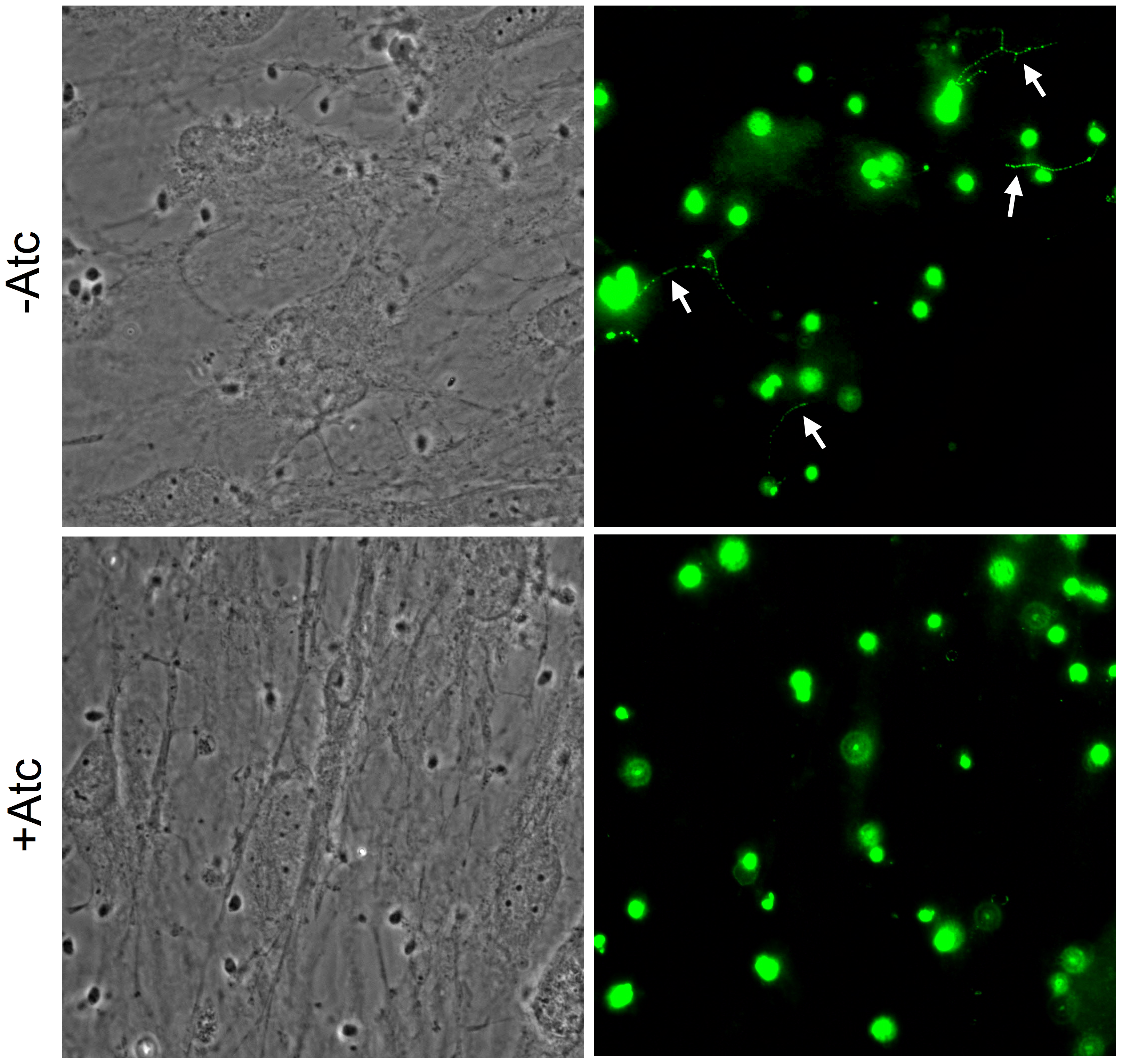

Supplement: Figure S1 — RON5 is critical for evacuole formation. IFA visualization of evacuole production by RON5cKD parasites. Representative images are shown for parasites with or without Atc treatment and individual evacuole trails are indicated (arrows). A dramatic decrease in evacuoles is seen following depletion of RON5. Results are quantified in Figure 3D. Green: mouse anti-ROP2/3/4 antibody detected by Alexa488-anti-mouse IgG. (TIF) [file ppat.1004025.s001.tif]

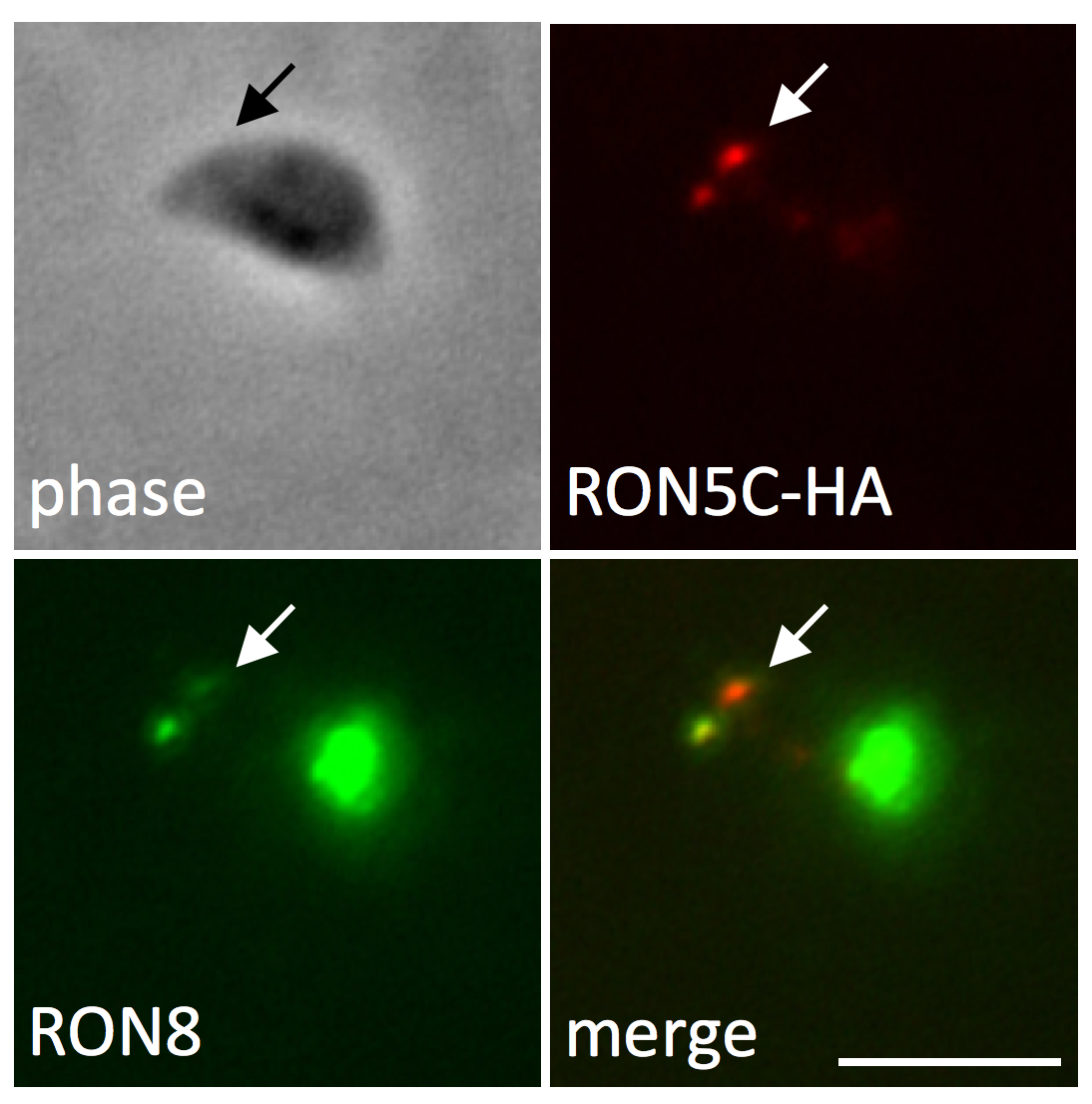

Supplement: Figure S2 — Rare invading RON5cKD parasites following Atc treatment always show visible levels of RON5. IFA showing a representative example of an invasion event by a RON5cKD parasite following 72 hours of Atc treatment. Note that while RON8 is robustly detected in the rhoptry necks, RON5 is barely detectible in the rhoptry neck (although clearly detected in the MJ - compare with untreated invading parasite shown in Figure 2D). Such invasion events were rare and were always accompanied by visible levels of RON5 in the MJ (arrows) and/or rhoptry necks. We visualized >100 invasion events across multiple experiments that all scored positive for RON5. This indicates that these rare invasion events are the result of residual RON5 that persists even after several days of treatment with Atc. Red: rabbit anti-HA antibody detected by Alexa594-anti-rabbit IgG. Green: mouse anti-RON8 antibody detected by Alexa488-anti-mouse IgG. Scale bar = 5 µm. (TIF) [file ppat.1004025.s002.tif]

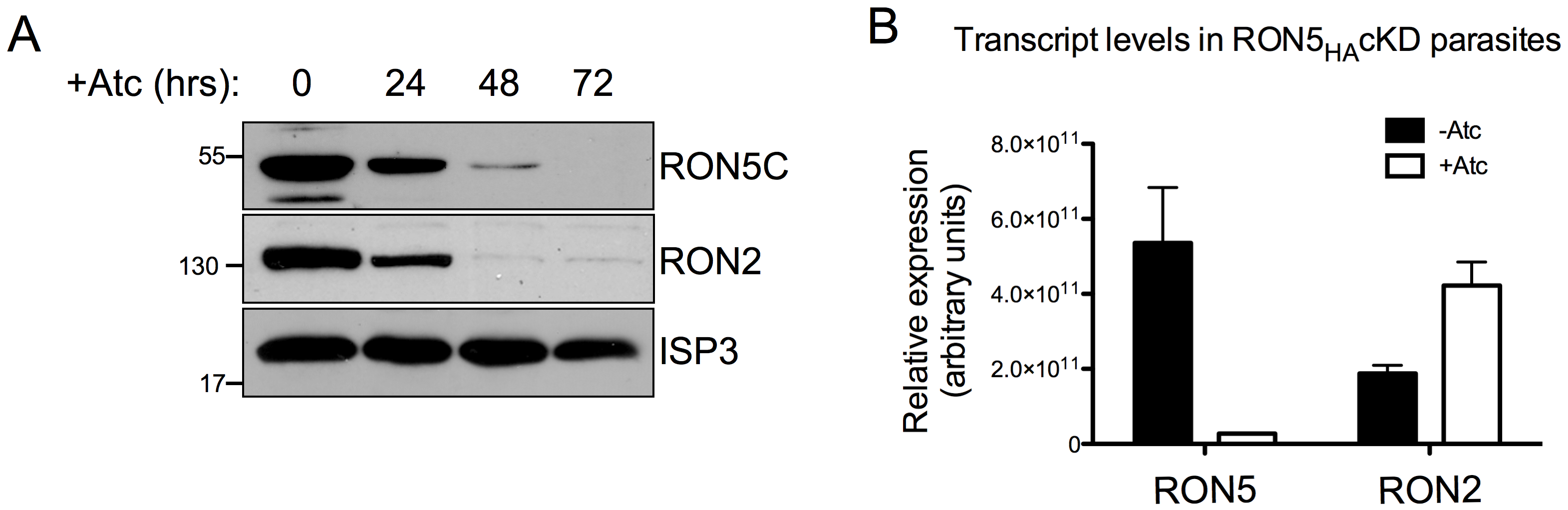

Supplement: Figure S3 — RON2 levels closely mimic RON5 levels during RON5 knockdown and destabilization of RON2 occurs at the protein level. (A) Western blot showing RON2 and RON5C levels after 24, 48 and 72 hours of Atc treatment. RON2 levels closely mimic diminishing RON5C levels showing that RON2 stability is dependent upon RON5. (B) qPCR analysis of RON5 and RON2 mRNA levels normalized to actin following 72 hours with or without Atc treatment. While a 19-fold decrease in RON5 transcripts is observed after Atc treatment, RON2 mRNA levels are not decreased and in fact show a small (∼2-fold) but reproducible increase. Data are representative of two independent experiments. (TIF) [file ppat.1004025.s003.tif]

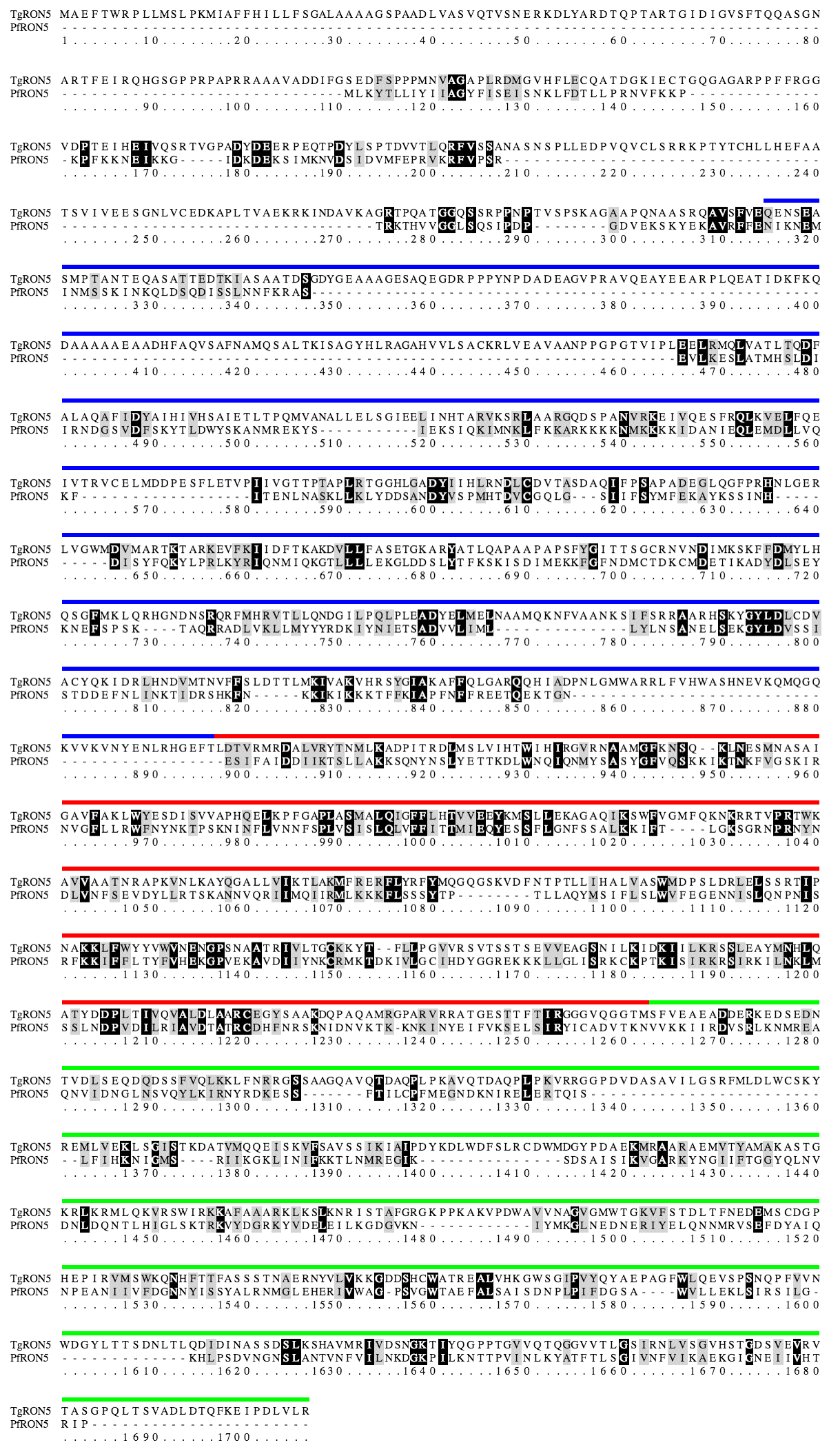

Supplement: Figure S4 — Alignment of RON5 orthologs. Alignment showing the level of conservation of RON5 sequence features between Toxoplasma and P. falciparum (GenBank accession numbers ACY08774 and ADV19051, respectively). Three general regions of differential conservation are seen: the highest level of conservation corresponds with the C-terminal portion of TgRON5N (residues 897–1257, red line) while the N-terminal portion of TgRON5N (residues 315–896, blue line) corresponds with a region of middle level conservation and TgRON5C (residues 1258–1702, green line) corresponds with a region of low conservation. The most N-terminal portion of TgRON5N as well as proTgRON5 show very low conservation with PfRON5. Alignment was generated using ClustalX and displayed using BoxShade (http://mobyle.pasteur.fr/cgi-bin/portal.py?#forms::boxshade). (TIF) [file ppat.1004025.s004.tif]

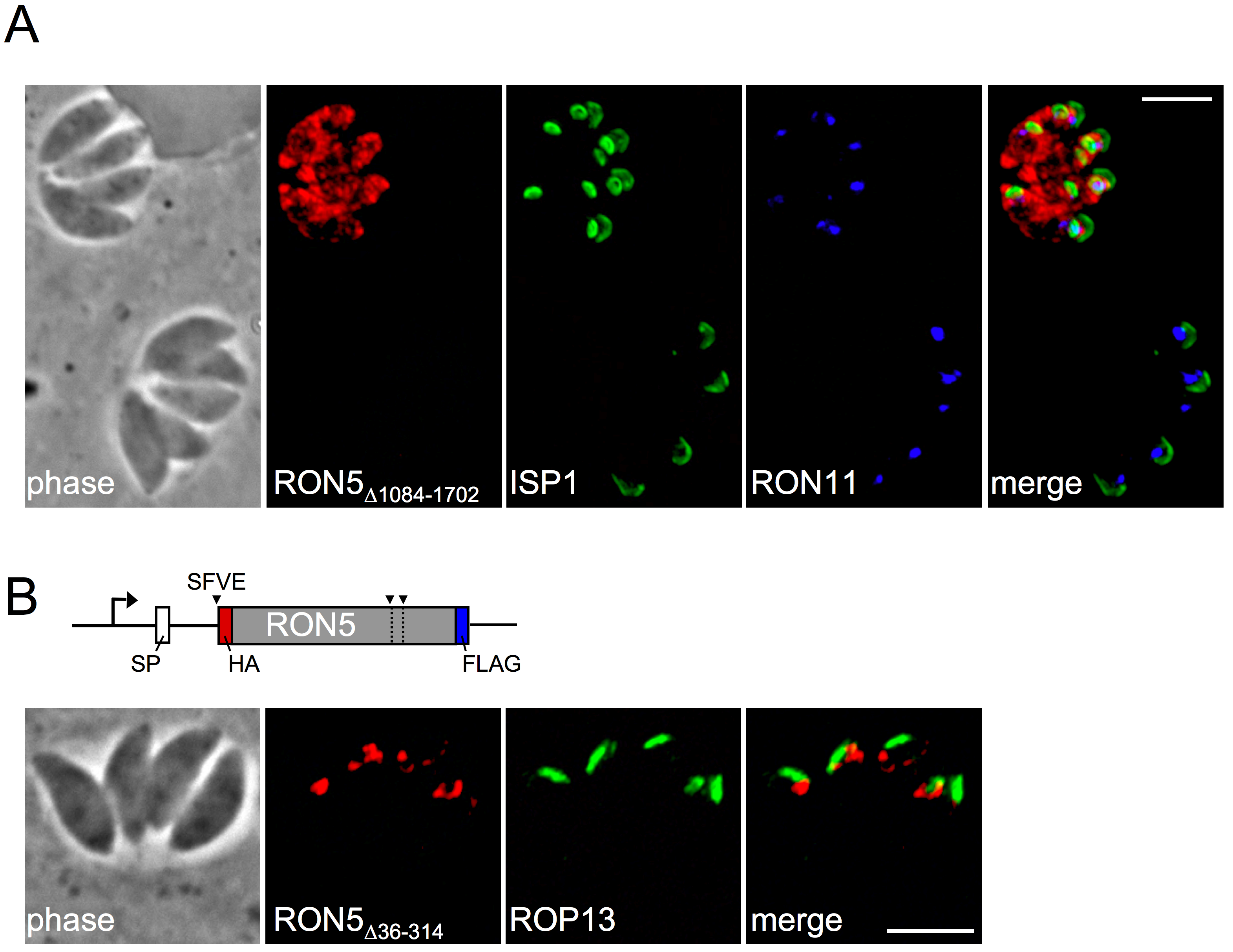

Supplement: Figure S5 — Analysis of mistargeted RON5 mutants by IFA. (A) Cell cycle variance of RON5 truncation mutant signal. RON5Δ1084-1702 was detected in cells in the process of assembling daughter parasites (upper vacuole, note ISP1 labeling of two daughter IMC apical caps within each parasite in addition to the maternal apical cap signal) but not in non-dividing cells (lower vacuole, only maternal IMC apical cap signal is seen). The same phenomenon was also seen for other C-terminal truncation mutants (data not shown). Red: rabbit anti-HA antibody detected by Alexa594-anti-rabbit IgG. Green: anti-ISP1 mAb 7E8 detected by Alexa488-anti-mouse IgG. Blue: rat anti-RON11 antibody detected by Alexa350-anti-rat IgG. All scale bars = 5 µm. (B) Inframe deletion of proRON5 results in a failure to target to the rhoptry neck with signal accumulating in a region posterior to the rhoptry bodies, likely corresponding with the parasite Golgi. Gross mistargeting was also seen with staining for FLAG (data not shown). Red: mouse anti-HA antibody detected by Alexa594-anti-mouse IgG. Green: rabbit anti-ROP13 antibody detected by Alexa488-anti-rabbit IgG. (TIF) [file ppat.1004025.s005.tif]

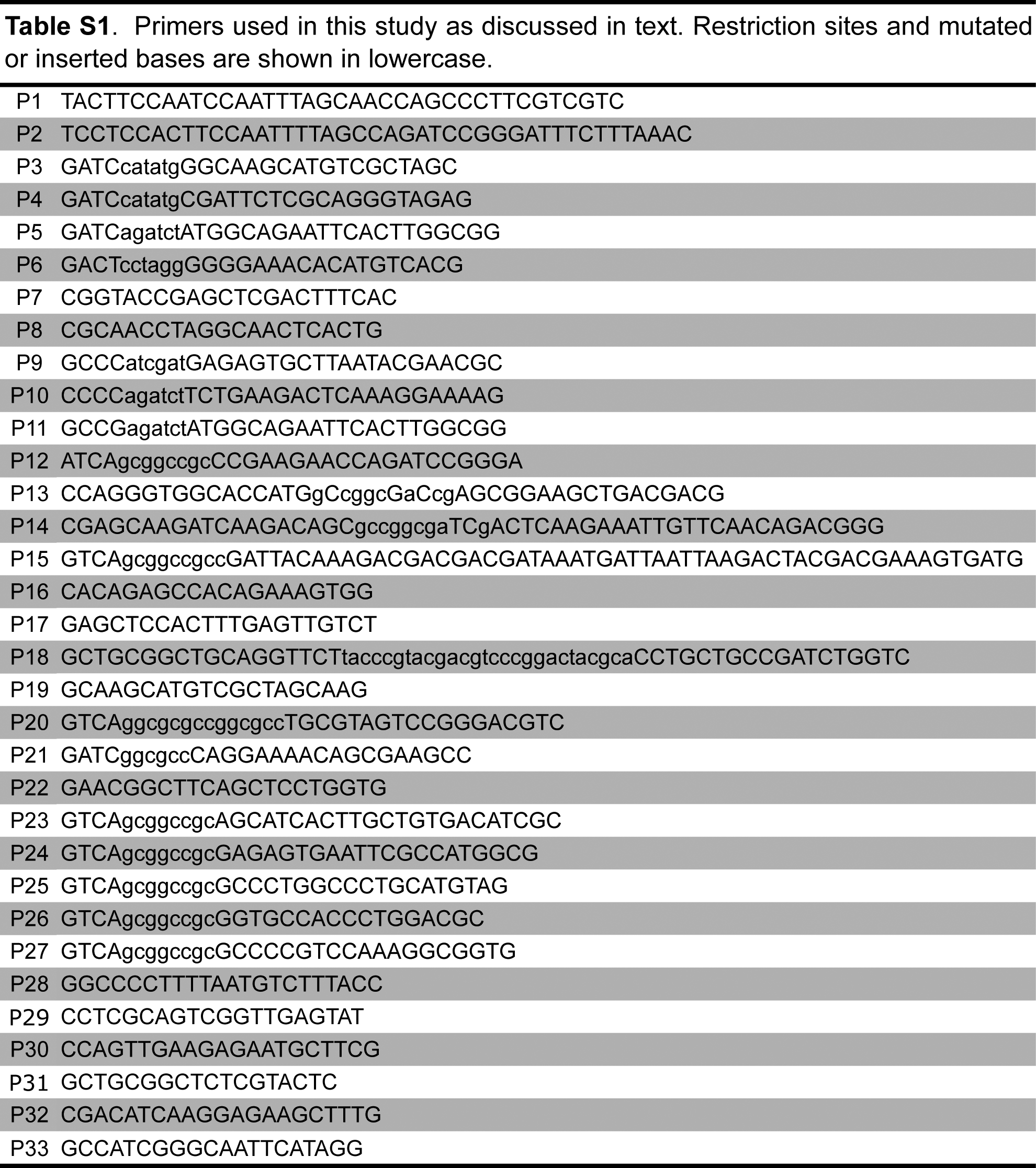

Supplement: Table S1 — Primers used in this study as discussed in text. Restriction sites and mutated or inserted bases are shown in lowercase. (TIF) [file ppat.1004025.s006.tif]
